# Neuroimmune disorders in COVID-19

**DOI:** 10.1007/s00415-022-11050-w

**Published:** 2022-03-30

**Authors:** Helena Ariño, Rosie Heartshorne, Benedict D. Michael, Timothy R. Nicholson, Angela Vincent, Thomas A. Pollak, Alberto Vogrig

**Affiliations:** 1 Institut d’Investigacions Biomèdiques August Pi i Sunyer (IDIBAPS), Barcelona, Spain; 2 Department of Psychosis Studies, Institute of Psychiatry, Psychology and Neuroscience, King’s College London, London, UK; 3 Department of Neurology, The Walton Centre NHS Foundation Trust, Liverpool, UK; 4 The National Institute for Health Research Health Protection Research Unit for Emerging and Zoonotic Infections, University of Liverpool, Liverpool, UK; 5 Department of Clinical Infection Microbiology and Immunology, Institute of Infection, Veterinary, and Ecological Sciences, University of Liverpool, Liverpool, UK; 6 Nuffield Department of Clinical Neurosciences, University of Oxford, Oxford, UK; 7 Centre de Référence National pour les Syndromes Neurologiques Paranéoplasique, Hôpital Neurologique, Hospices Civils de Lyon, Lyon, France; 8 Clinical Neurology Unit, Azienda Sanitaria Universitaria Friuli Centrale, Presidio Ospedaliero Santa Maria Della Misericordia, Udine, Italy

**Keywords:** Neuroimmunology, Autoimmune encephalitis, Limbic encephalitis, Guillain–Barre syndrome, SARS-CoV-2

## Abstract

Severe acute respiratory syndrome coronavirus 2 (SARS-CoV-2), the aetiologic agent of the coronavirus disease 2019 (COVID-19), is now rapidly disseminating throughout the world with 147,443,848 cases reported so far. Around 30–80% of cases (depending on COVID-19 severity) are reported to have neurological manifestations including anosmia, stroke, and encephalopathy. In addition, some patients have recognised autoimmune neurological disorders, including both central (limbic and brainstem encephalitis, acute disseminated encephalomyelitis [ADEM], and myelitis) and peripheral diseases (Guillain–Barré and Miller Fisher syndrome). We systematically describe data from 133 reported series on the Neurology and Neuropsychiatry of COVID-19 blog (https://blogs.bmj.com/jnnp/2020/05/01/the-neurology-and-neuropsychiatry-of-covid-19/) providing a comprehensive overview concerning the diagnosis, and treatment of patients with neurological immune-mediated complications of SARS-CoV-2. In most cases the latency to neurological disorder was highly variable and the immunological or other mechanisms involved were unclear. Despite specific neuronal or ganglioside antibodies only being identified in 10, many had apparent responses to immunotherapies. Although the proportion of patients experiencing immune-mediated neurological disorders is small, the total number is likely to be underestimated. The early recognition and improvement seen with use of immunomodulatory treatment, even in those without identified autoantibodies, makes delayed or missed diagnoses risk the potential for long-term disability, including the emerging challenge of post-acute COVID-19 sequelae (PACS). Finally, potential issues regarding the use of immunotherapies in patients with pre-existent neuro-immunological disorders are also discussed.

**Supplementary Information:**

The online version contains supplementary material available at 10.1007/s00415-022-11050-w.

## Introduction

Severe acute respiratory syndrome coronavirus 2 (SARS-CoV-2) is the aetiologic agent of the coronavirus disease 2019 (COVID-19). Since the earliest days of the pandemic, there have been many reports of central and peripheral neurological disease associated with the infection. Estimates of the frequency of neurological symptoms range from 30% patients admitted with confirmed COVID-19 [1] to 85% in patients in ICU or with acute respiratory distress syndrome (ARDS) [2–4]. Acute neurological complications are not only common, but they increase the short- and long-term burden of COVID-19 illness. For example, encephalopathy was independently associated with higher mortality within 30 days of hospitalization [1], and increased incidence of neuropsychiatric disorders within the 6 months after a COVID-19 diagnosis [5], raising the concern whether it is also an important risk factor for the neurological manifestations of the multi-organ syndrome PACS. While initial descriptions [6] were suggestive but unable to confirm a direct causal link between infection and neurological illness [7], an emerging epidemiological literature has shown that COVID-19 infection does appear to drive at least some of the neurological manifestations in the acute and long-term phase of the disease.

What is less clear is how these occur. Although in vivo animal studies have demonstrated the neurotropism of SARS-CoV-2, human studies do not support viral invasion of the nervous system as a major contributor [8]. Instead, a wide range of indirect mechanisms, often involving “immune dysregulation”, may converge in neurological dysfunction or damage. The cytokine storm triggered by the SARS-CoV-2 drives the delayed COVID-19 severity and it is likely involved in many of its neurological complications. However, following early reports of disorders such as Guillain–Barre syndrome (GBS) in the context of COVID-19 infection, studies have pointed to the development of neuronal autoimmunity as a potential important mechanism. Autoimmune neurological disorders have been associated with preceding viral infections in the past. From GBS, the prototypic post-infectious autoimmune disease, to neurological complications of influenza, Zika, and herpes simplex virus type one (HSV-1) infection, the latter associated with subsequent development of *N*-methyl-d-aspartate receptor antibody (NMDAR-antibody) encephalitis, many cases of postinfectious autoimmune aetiologies have been documented. These disorders may be associated with antibodies directed against self-antigens or tissues, that are thought to induce pathology at the level of the neuronal synapse, neuromuscular junction or myelin sheath [9–12]. In the context of COVID-19, however, the attribution of causality may be particularly difficult, requiring demonstration of two causally relevant processes (primary infection and secondary autoimmunity), often in the face of limited clinical and paraclinical information and overstretched healthcare resources. Determination of an immune-mediated aetiology is, however, crucial since it will likely mandate a distinct, frequently immunosuppressive, treatment approach. In this study we aimed to review the published cases of neuroimmune diseases described in association with COVID-19, assess the strength of this association, fulfilment of neurologic diagnostic criteria as well as clinical specificities, in order to provide guidance for the diagnosis and management of these emerging conditions.

## Methods

We aimed at the following : (1) define the clinical and paraclinical characteristics and management of immune-mediated neurological complications of COVID-19. For that we performed a systematic review of the literature using a regularly updated Neurology and Neuropsychiatry of COVID-19 blog (https://blogs.bmj.com/jnnp/2020/05/01/the-neurology-and-neuropsychiatry-of-covid-19/) as source; and (2) describe the available evidence on the management of patients with pre-existing neuro-immunological disorders in the COVID-19 setting. Participants of the study were probable or confirmed cases of SARS-CoV-2 infection (WHO criteria: WHO/2019-nCoV/Surveillance_Case_Definition/2020.2) who developed immune-mediated neurological complications, that were further categorized using internationally accepted diagnostic criteria [13–15]. Only studies prior to 26/04/2021 with ≥ 5 participants were included. In addition, studies describing antibody-positive cases were included irrespective of the number of patients considering their relevance in understanding the mechanisms of these disorders. Complete details of the methods are available in the Supplementary Material.

## Results

### Pathogenesis of neuroimmune disorders

**Fig. 1:**
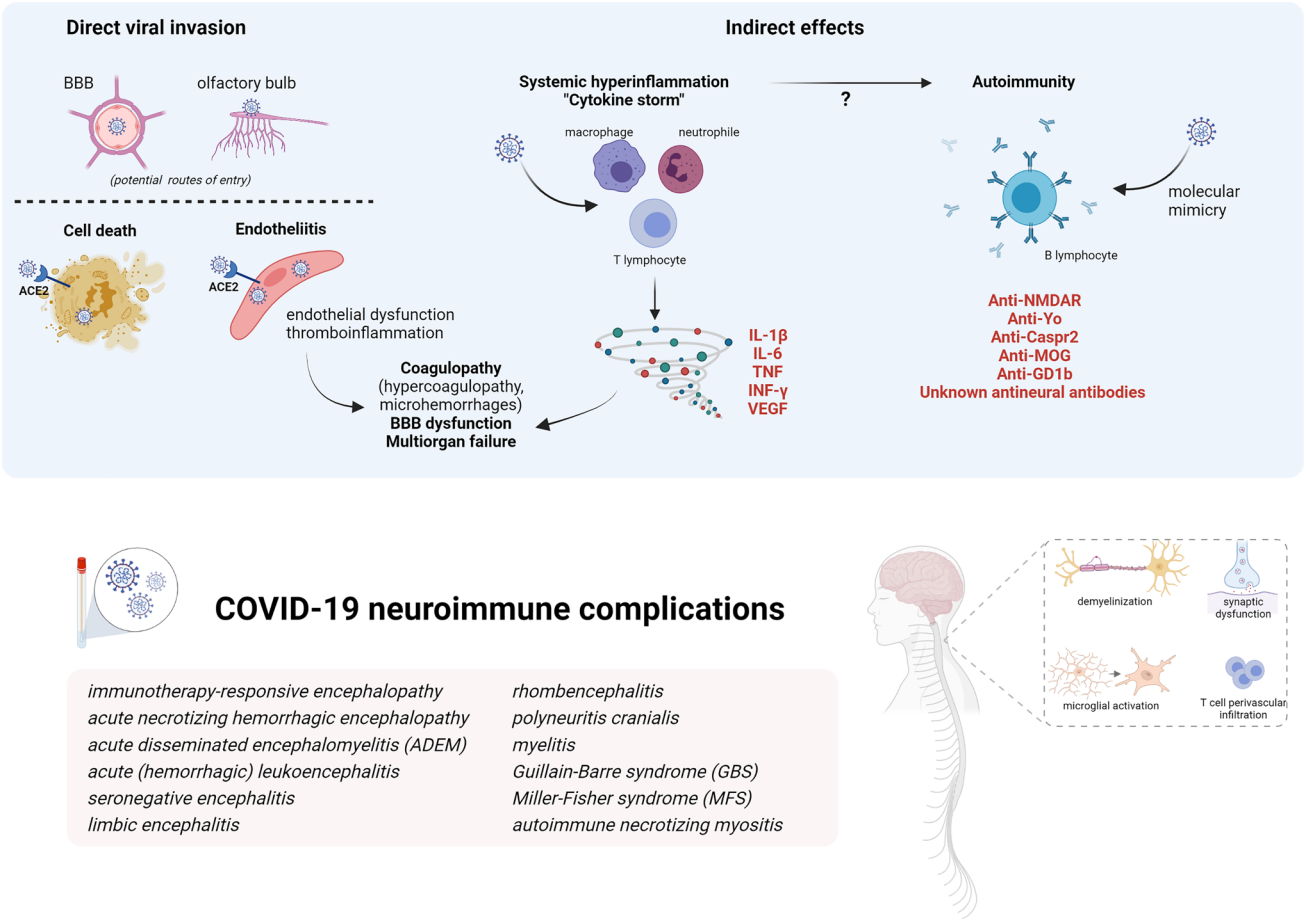
Mechanisms of SARS-CoV-2 pathogenicity and immune-mediated effects on nervous system (Created with BioRender.com)

Mechanisms of SARS-CoV-2 pathogenesis in the nervous system are diverse (Fig. 1) and include direct or indirect effects. These mechanisms are not mutually exclusive and might act synergically.

Potential routes of entries in the CNS for a direct viral invasion are by retrograde axonal transport from the olfactory system, by crossing the BBB or carried by infected immune cells. SARS-CoV-2 spike protein binds to angiotensin converting enzyme-2 (ACE2) for internalization, although other surface proteins may function as a co-factor. ACE2, a surface protein of many cell types, is highly expressed in the choroid plexus and found in neurons and astrocytes, oligodendrocytes, and in endothelial cells. Direct invasion of the virus can result in cell death or inflammatory infiltration of activated neutrophils and macrophages when invading endothelial cells (endotheliitis) which ultimately result in endothelial cell damage and thromboinflammation. Necropsy studies found activation of microglia and infiltration of cytotoxic T lymphocytes in brain parenchyma in some COVID-19 patients, suggestive of immune-mediated encephalitis. Among indirect effects, it has been demonstrated that SARS-CoV-2 is a potent trigger of innate and adaptive immune activation leading to overproduction of inflammatory cytokines, soluble mediators, hyperinflammation and multiorgan failure. Serum cytokine levels that are elevated in patients with COVID-19-associated cytokine storm include interleukin-1β, interleukin-6, IP-10, TNF, interferon-γ, macrophage inflammatory protein (MIP) 1α and 1β, and VEGF. This cytokine release syndrome may contribute to many of the clinical and laboratory findings reported in severe COVID-19: cytopenias, coagulopathy, hyperferritinemia and other acute-phase reactants (e.g., CRP, D-dimer) increase, endothelial damage and vascular permeability. In the brain, these cytokines can compromise the BBB and trigger a local amplification by inducing an innate immune response in resident cells which express toll-like receptors. Regarding autoimmunity, several antibodies against neuronal, glial or extraneural tissue are increasingly being described in both acute and recover in COVID-19 cases. The list of neuronal antibodies in the figure correspond to those exceptional COVID-19 cases described. It has been hypothesized that cross-reactivity due to molecular mimic could be the mechanism triggering this autoimmunity based on the molecular system involving gangliosides used by SARS-CoV2 to interact with the host cells and the detection of antiganglioside antibodies in cases of GBS after COVID-19.

#### Viral invasion

Direct effects consist of the SARS-CoV-2 nervous system invasion [8]. Although there is in vitro evidence of direct nervous system invasion and subsequent neuronal death, human neuropathological data have not yielded conclusive evidence of this mechanism so far and the most frequent histopathological findings are brain oedema, microthrombi, fresh ischaemic lesions and intense astrogliosis [8, 16]. Endothelial cell invasion and inflammatory infiltration, conversely, might be more relevant in pulmonary and extrapulmonary manifestations of COVID-19 [17]. Necropsy findings compatible with encephalitis do not correlate with the presence of virus in the brain; instead, they resemble the findings of immune-mediated encephalitis, predominantly in the brainstem and cerebellum [16].

#### Cytokine storm and autoimmunity

A major mechanism responsible for COVID-19 related severity is the ability to induce a systemic inflammation and cytokine storm following the initial replicative state [8, 18]. SARS-CoV-2 is a potent trigger of this immune hyperactivation which was initially described for certain systemic infections and well characterized for chimeric antigen receptor (CAR) T-cell therapy. High levels of cytokines can be detected in COVID-19 patients, both in serum and CSF, with worse prognostic associations and multiorgan failure. In particular, IL-6 might be a promising biomarker for severity and therapeutic decision-making, as antagonizing the IL-6 directly or through the JAK-STAT pathway has demonstrated improved prognosis in hospitalised COVID-19 patients with hypoxia and systemic inflammation [19, 20]. This cytokine storm has some specific features for COVID-19, for example it is frequently accompanied by lymphopenia in contrast to other disorders.

It is likely that the cytokine storm increases permeability of the blood brain barrier to potentially pathogenic circulating proteins (e.g. antibodies, other mediators) or to allow the systemic immune system to react aggressively against otherwise protected CNS antigens; unfortunately, data on blood brain barrier function is seldom available. It is also unknown whether the immune dysregulation associated with the cytokine storm contributes to some extent to activation and proliferation of autoreactive T cells initiating autoimmunity, the core pathophysiological mechanism underlying postinfectious disorders. Indeed, on the humoral arm of the immune system, multiple cases of potentially pathogenic CNS-targeting antibody responses have been described in association with both known and as yet uncharacterized neuronal or glial reactivities in COVID-19 patients. In one study, neutralizing human anti-SARS-CoV-2 antibodies showed cross-reactivity to unfixed murine tissue including brain, smooth-muscle, heart, lungs, kidney and colon [9]. Another clinical study found unexpectedly high rates of neuronal and glial antibodies in 11 patients presenting with varied neurological manifestations alongside COVID-19 infection [10]. Furthermore, potentially pathogenic antibodies to non-neuronal antigens, including antiphospholipid antibodies [11] are increasingly being described in both acute and recovered COVID-19 cases and have been variously associated with the severity of disease and outcome [12].

However, attribution of causality to self-reactive antibodies in patients with possible autoimmune neurological disease is far from straightforward, and even more challenging within a para-/postinfectious context. Evidence of pathogenicity for those antibodies against unknown nervous antigens has yet to be proven, and replication of these findings by other reference centres is awaited. Indeed, most studies of biomarkers in CSF from patients with CNS neurological conditions show a pattern more suggestive of cytokine release syndrome than antibody-mediated encephalitis, with increased levels of soluble mediators produced by the innate immune system (IL-6, TNF-α) and glial markers (GFAP), but with absence of intrathecal IgG synthesis and normal levels of chemokines associated with B/T-cell recruitment (CXCL13) [21–23]. Nevertheless, there is a recent interesting study analysing CSF and blood from individuals with COVID-19 with neurological symptoms, which found compartmentalized, CNS-specific T cell activation and B cell responses including antineuronal reactivity supporting autoimmunity in neurological complications [24].

### Central nervous system (CNS) immune-mediated disorders

**Fig. 2:**
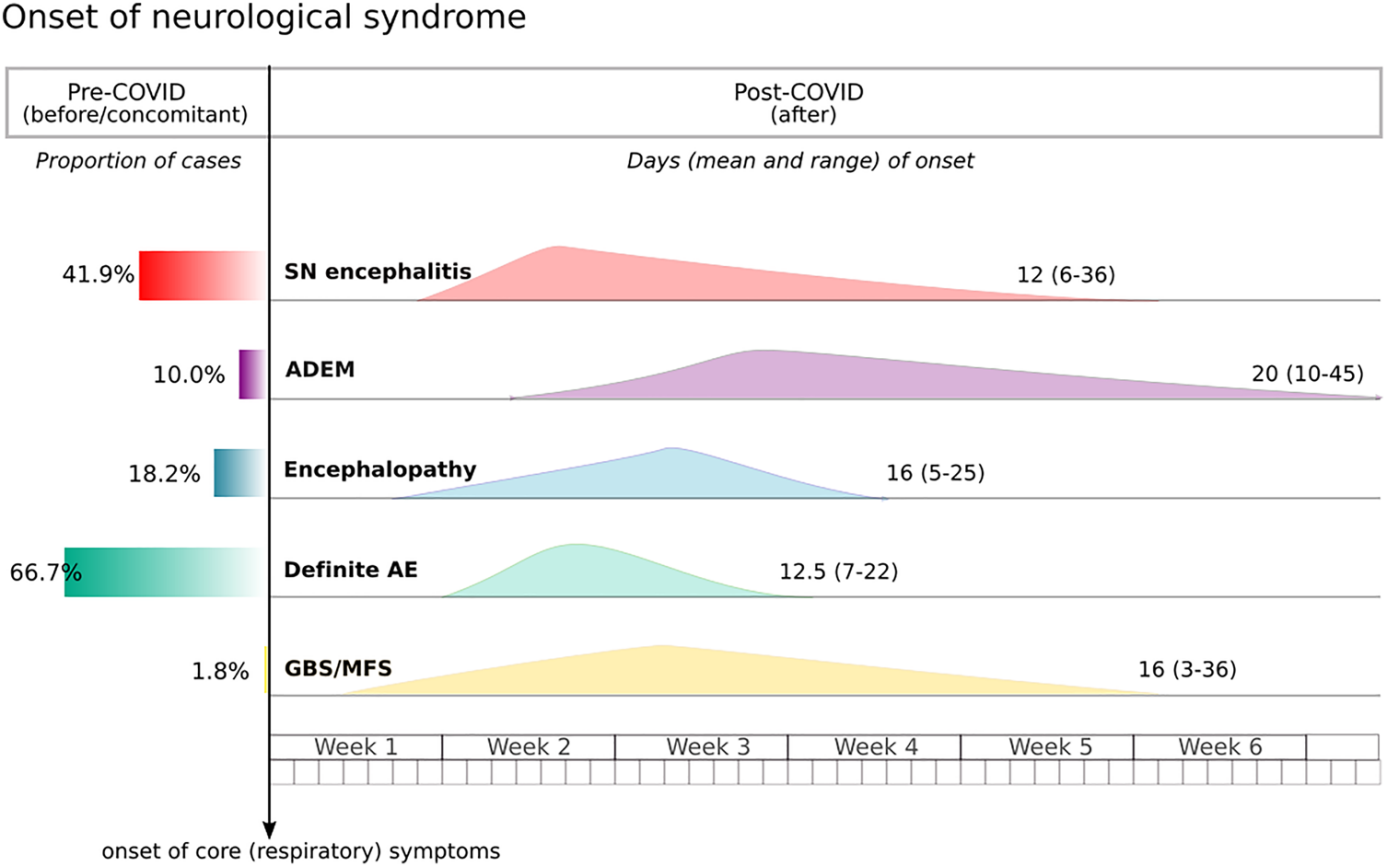
Timeframe of symptoms’ onset

Due to different operational diagnostic definitions and the limitations of comprehensive studies during the pandemic, the incidence of CNS immune-mediated disorders remains unknown. Nevertheless, the awareness of these neuroimmune complications has progressively grown during the pandemic. A systemic review published in July 2020 reported 8 cases of encephalitis among 901 (0.9%) COVID19 patients with neurological manifestations [25]. Conversely, in a recently published surveillance study of acute neurological and psychiatric complications of COVID-19 across the UK including 267 cases, 25 (9.4%) corresponded to inflammatory CNS disorders [26]. To date, however, fewer than 200 cases of immune-mediated CNS cases have been described. In this section, we analyse series of 5 or more patients [27–33]. These 64 patients are individually reported in Table S1, and compared to peripheral syndromes in Table 1. Figure 2 shows the temporal frame of neurological presentation in the COVID-19 evolution.

**Table 1:** Clinical and paraclinical characteristics of neurological immune-mediated disorders

	Encephalitis (seronegative)	ADEM/myelitis	Encephalopathy	Other definite AI encephalitis	GBS/MFS
*N* (included in this review)	43/133	10/133	11/133	12/133	57/133
Age, mean (range)	60 (22–77)	55 (48– ~ 74)	67 (51–78)	33 (2–80)	62 (23–77)
Female	17 (40%)	2 (20%)	5 (45%)	6/11 (55%)	17 (30%)
Neurological presentation after COVID-19, mean latency in days (range)	25 (58%), 12 (6–36)	9 (90%), 20 (10– ~ 45)	9 (82%), 16 (5–25 after intubation)	4 (33%), 12.5 (7–22)	55/56 (98%), 16 (3– ~ 36)
COVID-19 severity					
Respiratory asymptomatic	0	0	0	3 (25%)	0
Mild	2 (5%)	2 (20%)	1 (9%)	6 (50%)	9/43 (21%)
Mechanical ventilation	38 (88%)	8 (80%)	9 (82%)	1	12/43^a^ (28%)
CSF pleocytosis, other relevant findings	10/42 (24%), 1 OCB CSF-restricted	2 (20%)	0	8/11 (73%), 3 OCB CSF-restricted	0, 15/36 (42%) ACD
SARS-CoV-2 positive in CSF	0/37	0/7	0/8	2/10	1/11
Specific neuronal or ganglioside antibodies	0	0	0	10 (83%)	0/13
Received immunotherapy	27 (63%)	8 (80%)	11 (100%)	10/10 (100%)	54 (95%)
Clinical improvement after immunotherapy	16/27 (59%)	5/8 (63%)	9/10 (90%)	10/10 (100%)	34/45 (76%), 4 respiratory failure not COVID-19-related
Spontaneous improvement	8/43 (19%)	1/9 (11%)	0	0	n/a
Complete recovery	2/27	n/a	3	3	n/a
Death	7 (16%)	1 (10%)	1/10 (10%)	0	0

*AI* autoimmune, *GBS* Guillain–Barré syndrome, *MFS* Miller–Fisher syndrome, *OCB* oligoclonal bands, *ACD* albuminocytological dissociation

^a^Unknown contribution of GBS to respiratory failure among those 12 patients;  ~ estimated based on mean + 1 standard deviation from the original paper when it was not specified individually

This graph shows the presentation of neurological symptoms across the different syndromic groups in reference to major COVID-19 symptoms (namely respiratory and fever). The left panel represents the proportion of patients presenting neurological symptoms before or concomitant to COVID-19 disease onset. *SN* seronegative encephalitis, *AE* autoimmune encephalitis, *ADEM* acute disseminated encephalomyelitis, *GBS* Guillain–Barré syndrome, *MFS* Miller–Fisher syndrome.

#### Seronegative encephalitis

These cases had brain imaging or other evidence consistent with immune-mediated encephalitis and, apart from 9 patients diagnosed with ADEM described below, 43 patients (33% of total cohort; 17 females, mean [range 22–77] age of 60 years) met the criteria for autoimmune encephalitis based on clinical and paraclinical (MRI, CSF, EEG) criteria internationally accepted [13]. All of them were confirmed cases of COVID-19, and the presence of SARS-CoV-2 in the CSF was negative in all 38 that were tested. The neurological disorder occurred after COVID-19 presentation in 25 patients, with an approximate mean latency of 12 days from onset (range 6–36), but in the remaining 18 patients 16 presented simultaneously with COVID onset and in 2 patients the neurological disorder preceded it by 3–5 days [28]. Most patients had COVID-19 severe disease, and 38 required mechanical ventilation.

The most frequent neurological presentation reported was an acute brain dysfunction as encephalopathy or delirium, in 11 evident as prolonged weaning after sedation withdrawal. This was frequently accompanied by pyramidal signs and, cerebellar ataxia or brainstem dysfunction. One patient developed opsoclonus-myoclonus on top of confusion and hallucinations and another one presented a GBS concomitant to the encephalopathy. Nevertheless, combinations of psychiatric symptoms, prominent memory problems or seizures frequently described in non-COVID-related autoimmune encephalitis were not found in these series. Indeed, seizures (clinically or electrically defined) were reported only in 7 patients. Moreover, only 30 had some evidence of brain inflammation (20 suggestive MRI with normal CSF, 5 CSF pleocytosis with normal MRI and 5 with both abnormal).

Neuroimaging in these patients presents a practical challenge and the results further support a distinct type of encephalitis. A wide range of lesions were identified involving several brain areas. Only 3 of 43 patients had limbic encephalitis based on MRI (2 bilateral, 1 accompanied by diencephalic lesions). Other described lesions were confluent subcortical FLAIR/T2 hyperintensities, focal cortical and subcortical diffusion restriction, white-matter enhancing lesions, microbleeds, necrotic hemorrhagic lesions, and leptomeningeal enhancement. Leptomeningeal enhancement accompanied by bilateral frontotemporal hypoperfusion was one of the first imaging findings reported in 8/11 of patients admitted because of COVID-19-associated ARDS and neurological symptoms [34], but parenchymal abnormalities have been demonstrated to be more frequent in subsequent series. These lesions are located mainly in periventricular areas and centrum semiovale, but abnormalities in brainstem, cerebellar peduncles, basal ganglia, and corpus callosum were also described. A frequent radiological diagnosis was acute (haemorrhagic) leukoencephalitis, suggestive of a severe form of demyelinating disease [35]. A component of post–hypoxic leukoencephalopathy cannot be ruled out when symmetric confluent abnormalities without focal lesions predominate [30]. There were no bilateral thalamic lesions, hence no patient met criteria for the classic acute necrotizing encephalopathy described in other viral respiratory infections [14].

In contrast to the significant imaging findings of these patients, the CSF parameters were generally normal. Only 10/43 patients had pleocytosis, and 1 had unmatched oligoclonal bands. IL-6 was elevated in the CSF from 2 patients. Hyperproteinorrachia is slightly more frequent (60% in the ENCOVID Study)[28], but this is a nonspecific finding that can be present in metabolic entities such as diabetes mellitus. None of the 21/43 patients tested had neuronal autoantibodies when tested in serum or CSF. Other laboratory findings reported in 10 patients (either with critical or mild COVID19 disease) were high levels of peripheral inflammatory markers (CRP, ferritin, and/or D-dimer), in line with results described in a cytokine storm [18].

Eight patients showed spontaneous improvement and did not receive any specific treatment; 27 patients were treated with immunotherapy (11 steroids, 4 IVIG, 4 plasma exchange, 7 combinations of 2 of the above, 1 IVIG combined with Tocilizumab), after which 16 improved. Outcomes are not comparable since reported timepoints were very different (in some patients, a complete neurological recovery has been described, whereas in others ICU discharge is the reported outcome). Seven patients (16%) died, only one among those with normal MRI. Complete recovery was reported in only two patients.

#### Encephalopathy

Eleven patients (5 females, mean [range 51–78] age 67 years) did not meet possible autoimmune encephalitis criteria [29, 32, 36]. All of them were confirmed cases of COVID-19, 10 with severe disease. There was no evidence of SARS-CoV-2 in CSF in 8 patients tested. Neurological presentation was simultaneous with respiratory symptom onset in 1 patient, but in the other 10 there was a latency of days (range 5–25). The presentation consisted of failure to recover consciousness during the weaning period in eight patients and delirium with pyramidal signs in the rest. Neuroimaging was notable in five patients [36], showing signs of large cerebral arterial wall inflammation (abnormal contrast enhancement), without criteria of vasculitis, predominantly observed in the posterior circulation, similar to the pathological findings of endotheliitis reported in a previous post-mortem series [17]. CSF showed hyperproteinorrachia in 4 (none with signs of endotheliitis), all of them with an elevated CSF/serum albumin quotient suggesting blood–brain barrier dysfunction. Inflammatory markers (CRP, D-dimer, ferritin) and IL-6 were elevated in three patients’ sera. All of them were treated with immunotherapy: the five patients with endotheliitis had a rapid response after intravenous methylprednisolone, three showed complete recovery with IVIG (two combined with Tocilizumab and one with steroids), and plasma exchange was used in the three patients with post-intubation encephalopathy with different outcomes (one death and one still in the ICU but without respiratory support at the point of publication).

#### ADEM and transverse myelitis

Taking into account the diagnosis established by the authors of the original cases, [27, 30, 33] there were nine patients (two females, mean age 59 years) with acute disseminated encephalomyelitis and one case of transverse myelitis. Neurological presentation in ADEM occurred at a mean time of 23 days after COVID-19 disease onset in all patients except for one whose neurological disorder preceded COVID symptoms by 6 days. COVID-19 disease was severe in eight patients.

Besides demonstrating diffuse and large lesions involving predominantly the cerebral white matter, encephalopathy must be present to have a definite ADEM diagnosis [13]. This criterium was missing in the patient presenting with neurological symptoms before COVID-19, and the clinical picture described was more suggestive of an acute demyelinating polyradiculoneuropathy despite having CNS involvement on MRI. The main peculiarity of these case series is neuroimaging, which revealed abnormalities that are not typical for classical ADEM, showing necrosis or haemorrhages in 6/9 patients. On the other hand, CSF was not often informative. Only one patient had pleocytosis, no oligoclonal bands, and there was no evidence of intrathecal SARS-CoV-2.

Eight patients received immunotherapy (steroids, IVIG, or both) and five improved after treatment. One additional patient improved spontaneously. Most of them were gradually improving at the moment of publication, and one patient receiving only dexamethasone died.

#### Definite autoimmune encephalitis associated with SARS-CoV-2

To date, there are only 10 COVID patients with definite encephalitis associated with known neuronal antibodies 8 NMDAR-Ab [37–44], one CASPR2-Ab [45], and one MOG-Ab [46]. Table S2 provides details of these cases.

Contrary to seronegative encephalitis or ADEM patients, NMDAR-Ab patients were younger (mean age of 24 years, and two were children), symptoms occurred before or simultaneously to COVID in five patients, and COVID was generally mild or asymptomatic; MRI was normal (6) or showed unilateral mesotemporal hyperintensity (2). However, CSF analysis was more informative than in many of the COVID-related disorders: pleocytosis was found in six patients, and NMDAR antibodies were consistently detected in CSF. Diagnostic workup found an ovarian teratoma in one patient. These typical presentations and the absence of the temporality criterion raises doubts about SARS-CoV-2 being a cause of antibody-mediated encephalitis, but interestingly, SARS-CoV-2 RNA was detected in the CSF of two patients, thus raising the possibility of a parainfectious mechanism.

The CASPR2-Ab case was an 80-year-old patient without COVID-19 symptoms (but diagnosed by PCR) who presented with a 3-week neuropsychiatric syndrome in whom the work-up diagnosis revealed CASPR2-IgG antibodies both in the serum and CSF.

The MOG-Ab case was a 44-year-old female who presented with ADEM 7 days after a mild COVID-19 disease. MRI showed extensive demyelination with perivascular enhancement within the lesions, although no diffusion restriction or haemorrhage. This enhancement pattern was atypical for a classical ADEM, and the authors postulated that COVID-19-endotheliitis might be superimposed on the physiopathology of this case [17]. She showed rapid clinical improvement after immunotherapy.

There is one report of patients with undefined neuronal antibodies in patients with a variety of neurological syndromes (e.g. [10]) and a few case reports. One male developed malignant catatonia, with a normal MRI but pathological CSF (pleocytosis, oligoclonal bands and elevated IL-6), and IgG serum and CSF immunoreactivity to mouse brain, which improved after plasma exchange [47]. The same authors found CSF immunoreactivity from two females with MRI suggestive of encephalitis after COVID-19 onset, increased CSF IL-6 and good response to immunotherapy [48]. Finally, a young female with headache, drug-refractory seizures and neuroimaging suggestive of high-grade temporal glioma diagnosed after mild COVID-19 symptoms; biopsy showed concentric lymphocytic infiltration into perivascular spaces. [49]

### Peripheral nerve system (PNS) immune-mediated disorders

#### Guillain–Barre syndrome and Miller Fisher syndrome

To date, over fifty cases of GBS associated with the SARS-CoV-2 infection have been reported (Table S3) [50]. Most authors agree that the association here is causal rather than coincidental [51–53]. Filosto et al. reported a 2.6-fold increase in March–April 2020, compared with the preceding year [51]. Fragiel et al. also identified a higher prevalence of GBS in the SARS-CoV-2-positive population compared with the non-SARS-CoV-2 population attending the emergency department (0.15 versus 0.02%) [52]. However, agreement is not universal, with one large UK cohort study reporting a fall in cases of GBS March–May 2020, compared with the previous years [54]. Furthermore, they found no correlation between local incidence rates of SARS-CoV-2 and GBS. Nevertheless, the authors did agree that they could not exclude causality on a case-by-case basis. Additionally, they could not exclude a dip in overall GBS cases, due to lockdown and improved hand hygiene, masking a smaller increase due to SARS-CoV-2. This epidemiological discrepancy across different countries is interesting and is probably related to different methodologies adopted.

Cases of reported SARS-CoV-2-associated-GBS consistently fulfil the Brighton diagnostic criteria. The range of patients fulfilling the Brighton level one criteria is variable: 26.7% [51] and 55% [50], but this is not dissimilar to the proportion in the criteria validation study (41%) [15]. Furthermore, as many as 96.7% of cases were found to fulfil level one or two criteria [51, 52].

There are differences reported in latency between onset of SARS-CoV-2 infection and neurological symptoms (Fig. 2). Keddie et al. reported a latency between 0 and 37 days [54]. The mean latency in a systematic review by Sriwastava et al. 2021 was 12.5 days (SD ± 7.7) in the acute demyelinating polyradiculoneuropathy (AIDP) cohort and 11.1 (SD ± 4.9) in the acute motor axonal neuropathy and acute motor and sensory axonal neuropathy (AMAN/AMSAN) cohort [50]. By contrast, the largest single cohort study of SARS-CoV-2-associated-GBS had a mean latency of 24.2 days (SD ± 11.6) [51], which is a longer delay than that seen in other post-infectious GBS cohorts (median of 6 days after infection by Zika virus[55], 8–11 days after Varicella-zoster virus[56], and 11 days after Campylobacter jejuni[57]).

Clinical presentation in SARS-CoV-2-associated-GBS seems similarly distributed to ‘typical’ GBS. The majority exhibit a sensorimotor deficit in all four limbs associated with a demyelinating pattern on neurophysiology, in keeping with AIDP (66–77%) [51, 52, 58]. Rates of AMAN, AMSAN, and Miller Fisher Syndrome (MFS) also have similar distributions to non-COVID associated GBS. On the other hand, comparative studies between SARS-CoV-2-associated-GBS and non-SARS-CoV-2-associated-GBS cohorts have suggested a higher prevalence of cranial nerve (CN) involvement in the SARS-CoV-2 group [51, 54], with higher facial nerve involvement in 46.7% (20% bilateral), compared with just 17.6% in the non-SARS-CoV-2 cohort [51], as also were bulbar CN palsy (23.3% versus 5.9%) and oculomotor nerve palsy (10% versus 5.9%). A similar proportion of CN VII involvement (48%) was reported by Sriwastava et al.[50] 12% exhibited CN X palsy and 8% CN XII palsy. Whilst interesting, these studies are not designed or powered to determine statistically significant differences. There is some suggestion of higher rates of dysautonomia [51], but this is seldom looked at and may be attributed to a septic response to SARS-CoV-2 rather than pure dysautonomia. One other aspect is the absence of serum GM1, GD1a, or GQ1b ganglioside antibodies that are typically found in 53% of GBS patients but have been almost always negative in SARS-CoV-2 related patients, supporting the idea of a distinct cause.

Albumino-cytological dissociation (increased total protein with normal cell count) on CSF was demonstrated in 64% of cases of SARS-CoV-2-associated GBS and 85% of all cases had a WCC < 5 (100% < 50) [15, 53, 59–65]. When tested for CSF PCR testing for SARS-CoV-2, the majority were negative [62–72]; details of the one patient positive on CSF PCR were not available [52]. There has been a single case reported of positive SARS-CoV-2 antibodies in the CSF of a patient with GBS and raised CSF cytokines [73].

No clear differences in disease severity and treatment response between SARS-CoV-2-associated-GBS and ‘typical’ GBS were found, despite higher rates of ICU admissions and requirement for invasive ventilation [15, 51, 52, 54, 58]. However, it remains uncertain if this is due to higher rates of neuromuscular respiratory failure or secondary to the SARS-CoV-2 infection itself.

Treatment thus far has primarily been with intravenous immunoglobulins, in alignment with standard treatment of GBS [53, 61–63, 68, 70–72, 74, 75]. The majority of cases report partial or complete improvement [53, 62, 68, 71, 72, 74], but there have been some cases with continued deterioration or static neurology [61, 64, 70, 75]. Plasma exchange has been used less frequently and with variable outcomes.[64, 66, 76]. Finally, there have been a few isolated case reports of treatment with steroids, which conventionally do not improve outcomes in GBS [77], with different results [78–80].

The MFS subtype of GBS is characterised by the triad of ophthalmoplegia, ataxia, and areflexia [81], accounts for 5–25% of GBS cases, and can overlap with classic sensorimotor GBS and Bickerstaff brainstem encephalitis. To date, there have not been any striking differences in clinical presentation in the MFS cases described [59, 68, 82, 83]. However, whereas typical MFS strongly associates with GQ1b-Abs (86%) [78], these have not been detected in patients with any form of SARS-CoV-2-associated-GBS, except for one patient [59] who presented with a right intranuclear ophthalmoplegia, ataxia, and areflexia and had circulating GD1b IgG, a rarer antibody associated with better outcomes [84]. A second patient presenting with flaccid tetraplegia, progressing to bilateral facial palsies and neuromuscular respiratory failure, was positive for GM2 IgG and IgM [85]. This too is a much less commonly associated ganglioside antibody in GBS [86]. Despite being frequently tested for in SARS-CoV-2-associated-GBS cases, ganglioside antibodies have been negative, except for the aforementioned cases [60, 68, 69, 78, 79, 82]. The absence of typical post-infectious ganglioside antibodies supports a distinct disease mechanism in both SARS-CoV-2-associated-GBS and MFS.

### Pre-existing neuroimmunological disorders and risk of COVID-19

The COVID-19 pandemic raised relevant questions and concerns for neurologists taking care of patients with pre-existing neuroimmunological diseases treated with immunotherapy. Considering that it is well-established that certain drugs routinely adopted in neuroimmunology may increase the risk of infection by some pathogens, including viruses (e.g. John Cunningham virus [JCV] infection), an important question that needs to be answered is whether (i) the risk of infection by SARS-CoV-2 is different in patients taking immunomodulatory or immunosuppressant medications, and whether (ii) the risk of severe COVID-19 and death is the same as the general population in this selected group of patients [87–89]. In this context, clinical decision-making remains largely non-evidence-based, as few large-scale studies were published. The available evidence gathered from patients with neurologic and non-neurologic autoimmune disorders treated with immunotherapy as well as organ transplant recipients under immunosuppressant medications are overall reassuring insofar as there is no evidence of worse COVID-19 outcomes and major safety issues were not observed [87, 89, 90]. Several national and international recommendations (e.g. those from the Association of British Neurologists, ABN) [91] as well as opinion papers [92] have been published in order to guide the physician in managing immunotherapy during the pandemic. Most of these recommendations converge on the following points:

1. Ongoing immunotherapy should not be discontinued in clinically stable and non-lymphopenic neurological patients without COVID-19 infection, given the chance of neurological deterioration [87, 89, 92];
2. For patients with a relapse, corticosteroids, intravenous immune globulin (IVIG), and plasma exchange (PLEX) can be administered with a low risk. For patients under regular treatment with IVIG, switching to self-administered, subcutaneous IgG might be a reasonable alternative to limit exposure to hospital settings [92], since this approach was demonstrated to be non-inferior to intravenous administration [93].
3. Anti-CD20 agents (rituximab, ocrelizumab) showed an acceptable level of safety. Among patients receiving these drugs, those with advanced disability and older age are probably at higher risk of developing severe COVID-19 [88, 90], therefore prevention strategies should be reinforced in this particular group. Whenever the underlying neurologic condition is stable or improving, re-infusion could be delayed [94] or alternative options should be considered. In the management of patients with anti-CD20 therapy, depletion of B cells in the peripheral circulation lasts for at least 6 months after the last dose. The urgency of next infusion should be assessed by focusing on the clinical status, but additional help can be obtained by checking levels of peripheral B cells (B-cell repopulation is defined as 1% CD19+ cells of total lymphocytes) [87].
4. In patients with neuroimmunological diseases and severe COVID-19 requiring hospital admission, management of immunotherapy needs to be calibrated on an individual patient basis. In most severe cases, it appears reasonable to pause all injectables and oral immunosuppressant medications, while delaying infusions. This may not apply to all patients, and special considerations are related to corticosteroid use, as the RECOVERY trial showed that dexamethasone 6 mg p.o. daily for up to 10 days can be beneficial in patients with severe COVID-19 requiring oxygen support, but has no effect and should not be administered in cases with low or moderate severity [95]. Similarly, treatment with SARS-CoV-2 naïve IVIG has the potential to be beneficial given its action in reducing antibody-dependent hyperinflammation [87, 92], but should be used with caution in patient with high-risk of thrombosis (e.g. bedridden patients with sepsis in the ICU), since they can increase serum viscosity and COVID-19 patients are already at increased risk of thromboembolic complications, including stroke [96].
5. Overall, it appears that immunotherapy treatment and its modifications need to be personalized according to the severity of both neurologic condition and SARS-CoV-2 infection, taking into account also patient-related factors (including age and risk factors for severe COVID-19) and drugs’ pharmacokinetics [87]. Moreover, the effect of the same immunosuppressant can be different according to the stage of the disease (e.g. previous long-term treatment with corticosteroids can hamper the initial response to viral infection, while it can be beneficial during the stage of hyperinflammation and cytokine storm) [97]. Finally, it should be considered that some of the adverse events of these drugs can be masked or overlap with complications due to COVID-19, such as lymphopenia.

## Discussion and future directions

This review reveals several relevant clinical findings and some specificities as compared to the idiopathic counterparts. First, the commonest CNS manifestation is far from the classical autoimmune encephalitis picture associated with known neuronal antibodies, with an encephalopathy associated with severe COVID-19 disease the most frequent. Neuroimaging can show multifocal white matter abnormalities with haemorrhagic lesions, including ADEM-like presentations, while limbic encephalitis is rarely described. Neurological manifestations may follow respiratory ones, as expected in post-infectious conditions, but it is noteworthy that 42% of possible encephalitis in these series presented concomitantly with the respiratory symptoms, and only in ADEM was there a consistent delay before presentation. Neuronal antibodies were not found except in exceptional cases, and it is unclear if the SARS-CoV-2 is the trigger of the autoimmunity in these cases, as they generally lack a temporal latency and in some of them other known triggers have been demonstrated (teratoma). Spontaneous improvement is possible, response to immunotherapy is reported for first-line treatment (steroids, IVIG and plasma exchange) and there is no evidence to support chronic or more aggressive schemes of immunotherapy given the viral context.

Second, in GBS/MFS, clinical presentation and response to treatment overall do not indicate a clear distinct entity associated with SARS-CoV-2 and epidemiological studies are conflicting in demonstrating a causal link of SARS-CoV-2 and PNS although the absence of typical ganglioside antibodies indicates a distinct entity triggered by the viral infection. From a pragmatic point of view, this last clinical observation is relevant for the management of COVID-19 patients admitted to the ICU, where the development of subacute tetraparesis might not be always secondary to polyneuropathy and myopathy in the critically ill.

Third, although it is clear that empirical immunotherapy is beneficial in some COVID-19 patients with neurological complications, a better understanding of the pathophysiology to determine the subset of cases that is more likely to benefit from immunotherapy, if immunotherapy can prevent neuropsychiatric post-acute COVID-19 sequelae and which is the best therapeutic approach required. The diagnosis is also challenging, as in cases with CNS involvement criteria even for possible autoimmune encephalitis were lacking in 26% (20/76) of this aggregated series and definite criteria only in 16% (12/76, only 2 without antibodies). This highlights the need for novel biomarkers and further studies should explore in a more systematic way the utility of neuroimaging, CSF profiles including cytokines, and search for hitherto-unknown neuronal antibodies. Finally, for those patients with pre-existing neuroimmunological disorders treated with immunotherapy, the accumulated evidence has no raised safety concerns so far, but caution should prevail in the decision-making process.

## Supplementary Information

Below is the link to the electronic supplementary material.

Supplementary file1 (DOCX 51 KB)
