# Supplementary material for: Neuroimmune disorders in COVID-19
Source: J Neurol. 2022 Mar 30;269(6):2827–39. doi: 10.1007/s00415-022-11050-w (PMC9120100; doi:10.1007/s00415-022-11050-w)
Supplement: Supplementary file 1 — Supplementary file1 (DOCX 51 KB) [file 415_2022_11050_MOESM1_ESM.docx]

**Content:**

1. **Methods**
2. **Table S1.** CNS immune-mediated disorders
3. **Table S2.** Case reports of definite autoimmune encephalitis
4. **Table S3.** PNS immune-mediated disorders
5. **References**

**METHODS**

**Search Strategy and Selection**

This systematic review of the literature was performed using the resources developed by the CoroNerve Sudy Group until 28 April 2021, a UK-wide research initiative designed in collaboration with the Associations of British Neurologists (ABN) to systematically study the neurological and neuropsychiatric effects of COVID-19. Thanks to the partnership with the Journal of Neurology, Neurosurgery and Psychiatry (JNNP) a regularly updated blog (<https://blogs.bmj.com/jnnp/2020/05/01/the-neurology-and-neuropsychiatry-of-covid-19/>) was built and made freely available to collect relevant publications in a database using specified search strategies. We used the data collected in this database to retrieve papers describing immune-mediated neurological complications of SARS-CoV-2. References of the database were identified using the following search string in PubMed: (COVID-19 OR SARS-CoV2 OR SARS-CoV-2) AND (neurol* OR neuropath* OR nervous system OR brain OR encephal* OR meningit* OR stroke OR guillain-barre syndrome OR cerebr* OR psych* OR mania OR psycho* OR functional OR catatonia OR cognit* OR depress* OR anxi* OR obsessive OR post-traum* OR postraum* OR PTSD OR behaviour OR epilep* OR seizure OR headache* OR migraine OR crani* OR cloza* OR deliri*). In addition, considering that this is a rapidly moving topic, a search for relevant pre-prints was made by searching MedRxiv using the following string: COVID-19 OR SARS-CoV2 OR SARS-CoV-2 in ‘neurology’ and/or ‘psychiatry’ categories. Only studies with ≥ 5 participants, were included. In addition, studies describing antibody-positive cases were included irrespective of the number of patients in the study considering their relevance and potential impact in understanding the mechanisms of these disorders. Finally, national guidelines and expert opinion papers were reviewed in order to obtain guidance of the management of patients with pre-existing neurological disorders treated with immunotherapy during the COVID-19 pandemic.

**Participants**

Participants of the study were patients with documented infection by SARS-CoV-2 who developed immune-mediated neurological complications. Inclusion criteria were as follows: (1) neurological disease onset within 6 weeks of acute infection; (2) SARS-CoV-2 RNA detected in any sample or antibody evidence of acute SARS-CoV-2 infection; (3) no evidence of other commonly associated causes to explain the neurological dysfunction other than an immune-mediated disorder. Neurological disorders were categorized using internationally accepted diagnostic criteria. In detail, patients with suspected autoimmune encephalitis (AE) were classified according to the Graus criteria in possible, probable, or definite AE.[13] The criteria of acute disseminated encephalomyelitis (ADEM) specified in the same position paper were also adopted. Patients with acute necrotizing encephalopathy (ANE) were classified according to the Wong criteria,[14] which are based on neuroradiological features. Cases of Guillain-Barré syndrome (GBS) were categorized using the Brighton case definition.[15] Severity of COVID19 illness was defined as “mild-moderate” if there was no need for oxygen supply, “severe” when oxygen supply needed, “critical” when mechanical ventilation needed and “dead” for deaths secondary to COVID19.

**Table S1. CNS immune-mediated disorders (individual information when available)**

| **Ref** | **#‌ ‌patients,‌ ‌**  **age,‌ ‌sex‌** | **Neurological syndrome** | **Criteria AE** | **Latency (days)*** | **Clinical symptoms** | **MRI findings** | **CSF and other lab** | **COVID severity** | **Immunotherapy** | **Response and outcomes** |
| --- | --- | --- | --- | --- | --- | --- | --- | --- | --- | --- |
| Paterson et al. | 1, 65, F | Possible post-infectious encephalitis (presumed autoimmune) | possible | + 6 | Confusion, hallucinations, opsoclonus, stimulus sensitive myoclonus and convergence spasm, perseverance, bilateral ocular-facial synkinesis, dysarthria, orofacial apraxia | Normal | No pleocytosis. Serum: all neuronal antibodies performed were negative. Elevated CRP and D-Dimer, normal ferritin | severe | IVMP, oral prednisolone taper | Slow improvement but ongoing myoclonus; discharged home after 22 of admission |
| Pilotto et al. | 13, mean(sd) 66.5 (10.2), F(6) | Possible encephalitis MRI- | (no individual info) possible | -5 to -3 in 2, simultaneous in 7, mean(sd) +1.7 (5.6) in 4 | n/a | Normal | Pleocytosis (4/13), SARS-CoV-2 negative in CSF | critical | 4 (3 IVMP, 1 IVIg) | Response 3/13. Mean(sd) mRS 1.8(1.7), 1 death |
| Perrin et al. | 1, 53, F | Encephalopathy | possible | +7 approx | Headache at hospitalization, confusion, agitation, tremor, cerebellar ataxia, mild aphasia, behavioral alterations, cognitive disturbance. Acute kidney injury | Normal | No pleocytosis. Elevated CSF IL-6 in 2/5 patients from this series. SARS-CoV-2 negative in CSF. Serum: elevated CRP, IL‐6 and LDH. S100B protein elevated at the peak. Antineuronal antibodies were absent in all patients | critical | no immunotherapy | Spontaneous and gradual improvement until discharge on day 40 after symptom onset |
| Perrin et al. | 1, 67, M | Encephalopathy | possible | +7 approx | Headache, anosmia, dysgeusia at admission. Drop in visual acuity, VI cranial nerve palsy, cerebellar ataxia, behavioral alterations, pyramidal syndrome. Neurological manifestations occurred simultaneously with the peak in CRS serum markers. | Normal | No pleocytosis, OCB, elevated CSF IL-6 in 2/5 patients from this series. SARS-CoV-2 negative in CSF. Serum: elevated CRP, IL‐6 and LDH. S100B protein elevated at the peak. Antineuronal antibodies were absent in all patients | severe | steroids | Rapid improvement. On day 60, all neurological signs had regressed except slight palsy of left nerve VI (mRS 1) |
| Muccicoli et al. | 1, 67, M | Encephalopathy | possible | +20 | encephalopathy presented following sedation weaning as persisting impaired consciousness. Agitation, hemiparesis with pyramidal signs, extrapyramidal signs (rigidity and tremor), frontal release reflexes. Diffuse slowing EEG | Cerebral small vessel disease (chronic) | No pleocytosis, SARS-CoV-2 negative in CSF | critical | Tocilizumab 400 mg + IVIg | Complete neurological recovery 15 days after last IVIg |
| Pilotto et al. | 2, mean(sd) 72.0 (5.7), F(1) | LE | (no individual info) 1 definite seronegative AE, 1 probable | mean(sd) +14.0(1.4) | n/a | Bilateral limbic encephalitis (1), unilateral (1) | Pleocytosis (1/2), SARS-CoV-2 negative in CSF | critical | IVMP | Response 1/2 pt, mean(sd) mRS 1(1.4), 0 death |
| Pilotto et al. | 7, mean(sd) 63.6 (10.4), F(3) | Encephalitis | (no individual info) 3 probable | simultaneous in 4, mean(sd) +4.5(7.1) in 3 | higher prevalence of epileptic seizures than patients without abnormalities in MRI | multiple subcortical T2-hyperintensities (n=4), focal cortical T2 and DWI hyperintensities (n=3) and leptomeningeal enhancement (n=1) | Pleocytosis (3/7), SARS-CoV-2 negative in CSF | critical | 6 (4 IVMP, 2 IVIg) | No response to treatment, mean(sd) mRS 4.3(2.1) ; 3 death |
| Kihira et al. | 1, 59, F | Encephalopathy, hemorrhagic leukoencephalopathy | possible | simultaneous | acute disorientation on the day of presentation. There was no status epilepticus, discrete anoxic episode, or known toxic and/or metabolic abnormality to account for the imaging findings | Patchy and confluent areas of restricted diffusion and FLAIR hyperintensity in cerebral cortices and WM, (basal ganglia, splenium of corpus callosum, and pons). Scattered leptomeningeal FLAIR hyperintensity. Small subcortical and corpus callosum microhemorrhages. | No pleocytosis, negative OCB, SARS-CoV-2 negative in CSF | critical | currently undergoing plasma exchange | n/a |
| Kihira et al. | 1, 60, M | Encephalopathy, post–hypoxic leukoencephalopathy | possible | simultaneous | Encephalopathic on presentation, with disorientation and decreased alertness. His hospital stay was complicated by hypoxic respiratory failure, Takotsubo cardiomyopathy and acute renal failure. He continued to remain altered despite dialysis. Video EEG demonstrated diffuse theta slowing, but no epileptiform discharges. | Confluent FLAIR hyperintensity and diffusion restriction throughout the cerebral WM, with sparing of subcortical U-fibers. MR findings would be compatible with post–hypoxic leukoencephalopathy; however, because encephalopathy preceded respiratory distress, a component of COVID-19–related leukoencephalitis is also considered | No pleocytosis, SARS-CoV-2 negative in CSF | critical | n/a | n/a |
| Kihira et al. | 1, 35, F | Encephalopathy, hemorrhagic leukoencephalopathy | possible | simultaneous | 28 weeks’ gestation positive for COVID-19. Her hospital stay was complicated by hypoxic respiratory failure and cardiac arrest. Her mental status remained poor. EEG showed generalized slowing compatible with encephalopathy but no electrographic seizures | Numerous small foci of susceptibility within the peripheral cerebral subcortical WM and throughout the corpus callosum, most pronounced at the splenium, microhemorrhages.. Splenial confluent FLAIR hyperintensity was seen, consistent with edema; however, no other parenchymal edema | No pleocytosis, negative OCB, negative autoimmune antibodies | critical | n/a | n/a |
| Kihira et al. | 1, 41, M | Encephalopathy, post–hypoxic leukoencephalopathy | possible | +21 | Hospital course was complicated by hypoxic respiratory failure and acute kidney injury. The patient was on ventilation for 3 weeks and was found to be disoriented and decreased in alertness after extubation, which persisted despite several cycles of hemodialysis. | Symmetric FLAIR hyperintensity of the globi pallidi and more extensively throughout the cerebral WM, with subcortical U-fiber sparing, with corresponding moderately restricted diffusion. The leading consideration was delayed post–hypoxic leukoencephalopathy; however, basal ganglia involvement also prompted consideration of encephalitis | n/a | critical | n/a | n/a |
| Perrin et al. | 1, 71, F | Encephalopathy, acute leukoencephalitis | possible | simultaneous | Confusion, agitation, tremor, pyramidal syndrome, coma, dysautonomia, decerebration, death. Neurological manifestations occurred simultaneously with the peak in CRS serum markers | Symmetric FLAIR and DWI white matter hyperintensities predominantly in subcortical white matter suggestive of acute leukoencephalitis | No pleocytosis. Elevated CSF IL-6 in 2/5 patients from this series. SARS-CoV-2 negative in CSF. Serum: elevated CRP, IL‐6 and LDH. S100B protein elevated at the peak. Antineuronal antibodies were absent in all patients | severe | steroids | Temporary improvement after treatment, relapse, coma and death |
| Perrin et al. | 1, 64, M | Encephalopathy, acute leukoencephalitis | possible | simultaneous | Confusion, agitation, tremor, cerebellar ataxia, aphasia, apraxia, pyramidal syndrome, coma, dysautonomia. Neurological manifestations occurred simultaneously with the peak in CRS serum markers. | Acute leukoencephalitis and cytotoxic edema. FLAIR and DWI white matter hyperintensities in middle cerebellar peduncles, an acute mm‐scale cytotoxic edema on the posterior left frontal lobe, that persisted 16 days later excluding ischaemic stroke | No pleocytosis. Elevated CSF IL-6 in 2/5 patients from this series. SARS-CoV-2 negative in CSF. Serum: elevated CRP, IL‐6 and LDH. S100B protein elevated at the peak Antineuronal antibodies were absent in all patients | severe | steroids + IVIg | Improvement after CS, relapse, rapid improvement with IVIg. A second cycle of IVIg has been planned |
| Perrin et al. | 1, 51, M | Encephalopathy, acute hemorrhagic leukoencephalitis | possible | +7 approx | No neurological symptoms at admission. Confusion, agitation, tremor, cerebellar ataxia, pyramidal syndrome, behavioral alterations, cognitive disturbances. Severe COVID‐19‐related acute kidney injury | Acute hemorrhagic leukoencephalitis. FLAIR hyperintensities and micro‐hemorrhagic lesions in the splenium of the corpus callosum | No pleocytosis. Elevated CSF IL-6 in 2/5 patients from this series. SARS-CoV-2 negative in CSF. Serum: elevated CRP, IL‐6 and LDH. S100B protein elevated at the peak. Antineuronal antibodies were absent in all patients | critical | no immunotherapy | Spontaneous improvement. Ataxia and nystagmus improved partially, pyramidal syndrome and affective symptoms persisted on discharge on day 35 |
| Muccicoli et al. | 1, 69, M | Encephalopathy | possible | +23 | Encephalopathy presented following sedation weaning as persisting impaired consciousness. Agitation, tonic muscle spasms. Diffuse slowing EEG | Cerebral small vessel disease (chronic) | Pleocytosis, SARS-CoV-2 negative in CSF | critical | IVIg | Complete neurological recovery 1 day after last IVIg |
| Cao et al. | 1, 49, M | Encephalopathy | possible | +28 | Unresponsive wakefulness syndrome after sedation withdrawal. Brainstem impairment. Movement disorders | Deep hemispherical bilateral white matter lesions on T2/FLAIR with gadolinium enhancement on T1. Left posterolateral lesions of the pons on T2/FLAIR | No pleocytosis, negative OCB, normal IL-6 levels in CSF, SARS-CoV-2 negative in CSF. Serum: negative 'onconeuronal' antibodies | critical | IVMP + plasma exchange | Dramatic improvement 6 days after first PE session |
| Cao et al. | 1, 56, M | Encephalopathy, acute hemorrhagic leukoencephalitis | possible | +36 | Coma after sedation withdrawal. Brainstem impairment | Pontine tegmentum lesion on T2/FLAIR. Small hemorrhagic lesion of the left parietal lobe on SWAN. Multiple pontine microhaemorrhages within the tegmentum on SWAN | No pleocytosis, negative OCB, normal IL-6 levels in CSF, SARS-CoV-2 negative in CSF. Serum: negative 'onconeuronal' antibodies | critical | IVMP + plasma exchange | Dramatic improvement 2 days after first session. Transfer to rehabilitation center at 116 days (fully conscious, mild dysexecutive syndrome) |
| Cao et al. | 1, 61, M | Encephalopathy, acute hemorrhagic leukoencephalitis | possible | +23 | Unresponsive wakefulness syndrome after sedation withdrawal. Oculomotor disturbances, myorhythmias of the tongue and flaccid quadriplegia with facial diplegia associated with dysautonomia. Electroneuromyography at day 49 showed complete abolition of sensory and motor potential in four limbs, so COVID-19-related Guillain-Barre syndrome diagnosis was made. | Bilateral diffuse lesions of the deep subcortical white matter on T2/FLAIR. Multiple microhemorrhages of the corpus callosum on SWAN | No pleocytosis, negative OCB, normal IL-6 levels in CSF, SARS-CoV-2 negative in CSF. Serum: negative 'onconeuronal' antibodies | critical | IVMP + plasma exchange | Dramatic improvement 7 days after first session |
| Cao et al. | 1, 37, M | Encephalopathy, acute leukoencephalitis | probable | +33 | Unresponsive wakefulness syndrome. EEGs showed nonspecific diffuse slow-wave activity, unreactive, without any epileptic patterns | Several confluent periventricular and deep supratentorial white matter lesions on T2/FLAIR. Gadolinium-enhanced symmetrical bilateral focal lesions (centrum semiovale, pallidum periventricular white matter) on T1 | Pleocytosis, negative OCB, SARS-CoV-2 negative in CSF. Serum: negative 'onconeuronal' antibodies | critical | IVMP + plasma exchange | No improvement. Death |
| Cao et al. | 1, 77, F | Encephalopathy, necrotizing leukoencephalitis | possible | +32 | Unresponsive wakefulness syndrome. EEG at day 48 showed nonspecific diffuse slow-wave activity, inconstantly reactive, without any epileptic patterns | Several confluent hyperintense lesions in T2/FLAIR. sequence, located within the periventricular and deep supratentorial white matter. They mostly had necrotic centers, with slight peripheral gadolinium enhancement | No pleocytosis, negative OCB, normal IL-6 levels in CSF, SARS-CoV-2 negative in CSF. Serum: negative 'onconeuronal' antibodies | critical | IVMP + plasma exchange | No improvement. Death |
| Paterson et al. | 1, 66, F | Encephalitis, limbic-diencephlic encephalitis | possible | +1 | Confusion, single generalised seizure with low conscious level postictally lasting 48 hours, brisk reflexes, extensor plantars | T2 hyperintense signal changes in upper pons, both limbic lobes (symmetrical), medial thalami and subcortical cerebral white matter | No pleocytosis, OCB, elevated CSF proteins, viral PCR including SARS-CoV-2 negative. Serum: all neuronal antibodies performed were negative. Elevated D-dimer, CRP, normal ferritin | mild | IVMP, oral prednisolone taper + IVIg | Incomplete recovery, ongoing cognitive impairment, discharged home after 28 days of admission |
| Dogan et al. | 3, 49/59/22, M(3) | Encephalopathy | possible | 14-19 days after intubation | Critically ill with severe ARDS followed by a failure to recover consciousness or severe agitation during the weaning period | cortical or white matter hyperintensities, contrast enhancement, and sulcal hemorrhagic features, all of which are considered compatible with meningoencephalitis | No pleocytosis, OCB negative (2), elevated CSF proteins with increased Qalb (2). No autoimmune screening, nor CSF SarSCoV2. Serum: elevated DimerD, RCP, LDH, ferritin and IL-6 | critical | Plasma exchange 3-9 cycles | regained consciousness, extubation, ICU discharge |
| Pilotto et al. | 3, mean(sd) 65.3(9.5), M(3) | ADEM, 2 acute necrotising encephalitis |  | mean(sd) +32.0 (13.1) | n/a | Acute necrotising encephalitis in 2 (details missing) | Pleocytosis (1/3), SARS-CoV-2 negative in CSF | critical | 3 (2 IVMP, 1 IVIg) | response 1/3 pt, mean(sd) mRS 4(1.7), 0 death |
| Kihira et al. | 1, 48, M | ADEM, involving brain and spinal cord |  | +14 | COVID19 complicated by acute respiratory distress syndrome and acute kidney injury. The patient subsequently became altered, with flaccid paralysis of bilateral lower extremities. | Multiple diffusion-restricting and FLAIR hyperintense foci within the WM (right centrum semiovale, bilateral corona radiata, left temporal lobe, and left medullary pyramid), and in the spinal cord at C1. T2 FSE imaging showed a halo-like appearance of the lesion in the corona radiata and another, smaller lesion, which favored a demyelinating etiology. MRI spine: focal T2 hyperintense lesions at C1 and T5 levels as well as a third lesion in the left lateral column at T5 | No pleocytosis, OCB negative. | critical | n/a | n/a |
| Paterson et al. | 1, 52, M | ADEM, with hemorrhage |  | +22 | Slow to wake in ICU, hyperreflexia and clonus. Required renal replacement | Multiple clusters of lesions in the deep cerebral white matter. Cyst-like areas of varied sizes, some with hemorrhagic foci and peripheral rims of restricted diffusion | No pleocytosis. OCB negative, and viral PCR and antibodies negative in CSF. Serum: all neuronal antibodies performed were negative. Elevated D-dimer, ferritin, CRP | critical | no immunotherapy | Incomplete but progressive ongoing recovery |
| Paterson et al. | 1, 60, M | ADEM, with hemorrhage |  | +27 | Slow to wake in ICU, right extensor plantar | Multifocal and confluent areas of signal change in the cerebral hemispheric white matter with extensive microhemorrhages in the subcortical regions | No pleocytosis. CSF OCB negative, viral PCR negative including SARS-CoV-2. Serum: all neuronal antibodies performed were negative. Elevated D-dimer, ferritin, CRP | critical | IVMP | Incomplete but progressive ongoing recovery |
| Paterson et al. | 1, 59, F | ADEM |  | +10 | Recurrent fleeting episodes of vacant staring and speech arrest, generalised tonicclonic seizures, headache, low conscious level leading to prolonged intubation, left extensor plantar | Extensive, confluent and largely symmetrical areas throughout brainstem, limbic and insular lobes, superficial subcortical white matter and deep grey matter. Clusters of microhemorrhages, restricted diffusion and peripheral rim enhancement | No pleocytosis, viral PCR negative including SARS-CoV-2. | mild | Dexamethasone | No response. Death |
| Paterson et al. | 1, 52, M | ADEM with hemorrhage and AIDP | no criteria ADEM | -6 | Headache, back pain, progressive flaccid limb weakness (proximal > distal) with facial and neck weakness, areflexia; extensor plantars, normal sensation, ophthalmoplegia day 3. NCS - moderately severe acute demyelinating polyradiculoneuropathy | Multifocal confluent lesions in internal and external capsules, splenium and deep white matter of cerebral hemispheres. Over 5 days, lesions increased in size and showed multiple microhemorrhages. Components of brachial and lumbosacral plexus showed increased signal and enhancement | No pleocytosis. CSF protein raised, viral PCR negative including SARS-CoV-2. Serum: all neuronal antibodies performed were negative. Normal peripheral inflammatory markers | critical | IVMP + IVIG | Incomplete but progressive ongoing recovery |
| Paterson et al. | 1, 60, F | ADEM |  | +18 | Slow to wake in ICU, extensor posturing of limbs. EEG – diffuse encephalopathy. Required renal replacement | Multifocal lesions with diffusion changes in periventricular white matter and corpus callosum. | No pleocytosis, CSF protein raised, viral PCR negative, viral PCR negative including SARS-CoV-2. Serum: all neuronal antibodies performed were negative. Elevated D-dimer, ferritin, CRP | critical | IVMP, oral taper | Incomplete but progressive ongoing recovery |
| Paterson et al. | 1, 48, M | Post-infectious myelitis |  | +19 | Numbness of hands and feet, band of itching sensation at level of the umbilicus, sensory ataxia, brisk reflexes with extensor plantars, pinprick level to T10 | MRI thoracic spine showed a patchy area of intramedullary high signal in the dorsal cord primarily at T5-6 and T10-11 and down to the conus with no enhancement with contrast. MRI brain normal | Pleocytosis, CSF culture and viral PCR negative. Serum: all neuronal antibodies performed were negative. Normal peripheral inflammatory markers | mild | IVMP | Incomplete but progressive recovery. Ongoing rehabilitation |
| Muccicoli et al. | 1, 54, F | Encephalopathy | no criteria | +5 | Irritability, quadriparesis with pyramidal signs, akinetic mutism, agitated delirium, frontal release reflexes. Diffuse slowing EEG | Fronto-parietal white matter hyperintensity | No pleocytosis | critical | Tocilizumab 400 mg, low-dose steroids and IVIg | Complete neurological recovery last day of IVIg |
| Muccicoli et al. | 1, 75, M | Encephalopathy | no criteria | simultaneous | Confusion, disorientation, global memory deficits. Diffuse slowing EEG | Previous right fronto-parietal stroke | No pleocytosis, elevated CSF proteins with increased Qalb. SARS-CoV-2 negative in CSF | severe | Tocilizumab 400 mg, low-dose steroids and IVIg | Complete neurological recovery IVIg 4 days after last IVIg |
| Muccicoli et al. | 1, 69, F | Encephalopathy | no criteria | +15 | Apraxia, mixed delirium, pyramidal signs, frontal release reflexes, extrapyramidal signs (rigidity and bradykinesia). Diffuse slowing EEG | Parietal white matter hyperintensity, cerebral atrophy | No pleocytosis, SARS-CoV-2 negative in CSF | mild | IVMP + IVIg | Complete neurological recovery 6 days after last IVIg |
| Dogan et al. | 1, 59, M | Encephalopathy | no criteria | 14 days after intubation | Critically ill with severe ARDS followed by a failure to recover consciousness or severe agitation during the weaning period | Normal | No pleocytosis, OCB negative, elevated CSF proteins with increased Qalb. Serum: No autoimmune screening, elevated DimerD, RCP, LDH and ferritin | critical | Plasma exchange 1 cycle | Progression despite treatment. Death |
| Dogan et al. | 1, 51, F | Encephalopathy | no criteria | 14 days after intubation | Critically ill with severe ARDS followed by a failure to recover consciousness or severe agitation during the weaning period | Normal | No pleocytosis, OCB negative, elevated CSF proteins with increased Qalb. Serum: No autoimmune screening, elevated DimerD, CRP, LDH and ferritin | critical | Plasma exchange 5 cycles | Regained consciousness, extubation, ICU discharge. |
| Dogan et al. | 1, 55, M | Encephalopathy | no criteria | 25 days after intubation | Critically ill with severe ARDS followed by a failure to recover consciousness or severe agitation during the weaning period | Normal | No pleocytosis, OCB negative, elevated CSF proteins with increased Qalb. Serum: No autoimmune screening, elevated DimerD, CRP, LDH, ferritin and IL-6 | critical | Plasma exchange 5 cycles | Unknown response. Still on ICU due to CMV reactivation |
| Pugin et al. | 5, median 75 (IQR: 69-78), M (3) | Encephalopathy | no criteria | 14 days after intubation | Critically ill with severe ARDS followed by a failure to recovery consciousness (GSC 4-9) | Abnormal contrast enhancement of the vascular wall was noted in all  patients, predominantly in the basal skull arteries, without other vasculitis sings (endotheliitis) | No pleocytosis (except one patient with lymphoma), OCB type 4 (in four) and type 5 (in one), normal CSF protein content. SARS-CoV-2 negative in CSF | critical | IVMP | Regained consciousness (3/5), 2 patients with fluctuating variations of arousal |

* Positive values mean that neurological symptoms occurred after COVID19 disease onset

Abbreviations: ARDS, Acute Respiratory Distress Syndrome; CRP, C-Reactive Protein test; CSF, cerebrospinal fluid; EEG, electroencephalogram; ICU, intensive care unit; IVIg, intravenous immunoglobulins; IVMP, intravenous methylprednisolone; LE, limbic encephalitis; MRI, magnetic resonance imaging; mRS, Modified Rankin Scale; OCB, oligoclonal bands; Qalb, CSF/serum quotient of albumin; sd, standard deviation.

**Table S2. Case reports of definite autoimmune encephalitis (antineuronal antibodies or compatible biopsy)**

| **Ref** | **Age, sex** | **Syndrome** | **Criteria AE** | **Latency (days)** | **Clinical symptoms** | **MRI** | **CSF and other lab** | **COVID severity** | **Treatment NRL** | **Response and outcomes** |
| --- | --- | --- | --- | --- | --- | --- | --- | --- | --- | --- |
| Pinto et al. | 44, F | Encephalitis MOG-ab, ADEM | Definite AE | +7 | Over 48 hours, the patient developed word-finding difficulties and right arm weakness, deterioration over the next 6 days with the development of severe aphasia and hemiparesis. Repeat MRI brain scan, 6 days after presentation, showed progression of the bilateral centrum semiovale and white matter changes | T2-hyperintensity within the centrum semiovale bilaterally in a periventricular location, extending along the left temporal and occipital horns and into the subcortical deep white matter bilaterally, more extensive in the left hemisphere. Perivascular enhancement within the lesions, although no diffusion restriction, hemorrhage, or mass effect was found. Spinal MRI normal | Pleocytosis, negative OCB, SARS-CoV-2 negative in CSF. Serum: anti-MOG antibodies | mild | IVMP day 6, prednisolone 60 mg daily. Plasma exchange day 8 | Rapid improvement in the neurologic deficit after the patient started immunomodulatory treatment. Normal speech, almost full power in the right arm and leg, and no visual or sensory inattention at day 18 |
| Álvarez Bravo et al. | 30, F | Anti-NMDAR encephalitis, left LE | Definite anti-NMDAR encephalitis | -3 | 3-day history of behaviour disorder characterised by psychomotor agitation, paranoid ideation, dysarthria with dysprosody, and visual hallucinations. fever, and nasopharyngeal exudate was sampled to test for SARS-CoV-2 infection, without pneumonia. She presented several episodes of focal seizures and some generalised seizures. EEG revealed epileptic discharges in the left frontotemporal region. Despite steroids, decrease in the patient’s level of consciousness and buccolingual dyskinesia and choreo-dystonic movements of the right hand with delta brush EEG pattern. Left ovarian teratoma detected. ICU complications as hypovolemic shock and post-surgical intra-abdominal infection, as well as pneumonia, thrombosis of the left iliac vein, and bilateral pulmonary embolism attributed to SARS-CoV-2 infection | Hyperintensities in the left hippocampus | Pleocytosis, SARS-CoV-2 negative in CSF. Antibodies anti-NMDAR in serum and CSF | mild | IVMP x2, IVIg, teratoma resection (laparotomy), rituximab (delayed 1 month due to infectious comorbidities) | Neurological status remained unchanged until rituximab. 2 months later discharged from hospital with cognitive sequelae such as hypoprosexia, emotional lability, and memory disorder, and enrolled in an intensive neurorehabilitation programme |
| Burr et al. | 23, F | Anti-NMDAR encephalitis | Definite anti-NMDAR encephalitis | +7 | 1 week history of fever, fussiness, poor sleep, constipation, and decreased oral intake. At admission she was no longer talking and had nearly constant kicking and thrashing movements of her arms and legs. Two days after admission, she had several seizures, treated acutely with lorazepam and levetiracetam. Two weeks into the disease course, despite resolution of fever, she continued to have worsening encephalopathy and persistent hyperkinetic movements of the arms, legs, and head | normal | Pleocytosis, SARS-CoV-2 negative in CSF, antibodies anti-NMDAR in serum and CSF | mild | IVMP + IVIg | No immediate response to IVMP. Over the following week, gradually improvement. Returned to her baseline two weeks after being discharged from the hospital |
| Monti et al. | 50, M | Anti-NMDAR encephalitis (NORSE) | Definite anti-NMDAR encephalitis | simultaneous | Acute onset of psychiatric symptoms (confabulations and delirious ideas), fever without respiratory symptoms. After 4 days, focal motor seizures with impaired awareness and oro-facial dyskinesia/automatisms appeared. A first brain MRI was negative. He suddenly developed a refractory status epilepticus (RSE), despite treatment with diazepam, valproic acid and lacosamide, requiring admission to the intensive care unit (ICU) and anesthetics’ treatment. No sign of interstitial pneumonia. Negative tumour screening | normal | Pleocytosis, OCB, SARS-CoV-2 negative in CSF, antibodies anti-NMDAR in CSF (negative in serum), raised levels of IL6 and IL8 in CSF | mild | IVMP, IVIg, plasma exchange | After having completed the first cycle of plasma-exchange, seizures definitely ceased thus anesthetic therapy was stopped (day 47). Four months after the onset the patient was discharged at home in good condition, autonomous, and without neurological deficits |
| Panariello et al. | 23, M | Anti-NMDAR encephalitis | Definite anti-NMDAR encephalitis | -3 | psychomotor agitation, anxiety, thought disorganization, persecutory delusions and auditory hallucinations with commanding voices and global insomnia which appeared over three days, treated with haloperidol, promazine and intranasal midazolam and subsequently with intramuscular aripiprazole and oral quetiapine with no clinical response, followed by fever and desaturation. Bilateral pneumonia in CT. Oxygen therapy was necessary. Within the second week, the patient was found encephalitic, non-verbal, non responsive to commands. EEG showed theta activity at 6 Hz. Three weeks following hospital admission, the neurological symptomatology worsened despite off-label therapy based on hydroxychloroquine and darunavir/cobicistat, with severe dysphagia, dyskinesias, autonomic instabilities with wide ranging fluctuations in body temperature, blood pressure, respiratory rate and cardiac rhythm | n/a | Traumatic LP, SARS-CoV-2 negative in CSF, antibodies anti-NMDAR in CSF. Serum: increased IL-6 | severe | Dexamethasone + IVIg | "Clinical conditions are ameliorating to date" |
| Mulder et al. | 40, M | Malignant catatonia | Possible AE | +22 | Acute debut of agitation, grimacing, repetitive speech and movements (verbigeration and stereotypies) 24h. Behaviour was bizarre, disorganised, hyperkinetic and uncooperative. Fever at admission (COVID19 diagnosis 22 days before, dyspnoea and fatigue, which did not require hospitalization). Clinical progression in hours: mutistic, autonomic instability. Decorticate posturing, increased tonus, resisted movement of arms and jaw but had normal tonus in the legs. Bilateral hyperreflexia. Continuous EEG monitoring showed nonspecific slowing with left hemisphere predominance without epileptiform activity. Episode of asystole with spontaneous recovery and bradycardia of 27 bpm due to atrioventricular block III. On day 28, after the patient showed a dramatic improvement, became evident visual hallucinations, emotional lability and mental fatigue with disturbed short-term memory and decision making. FDG PET scan, day 35 (13 days after treatment initiation), showed high bilateral uptake in the striatum (caudate nucleus and putamen) | normal (x2) | Pleocytosis, elevated IgG index, OCB, SARS-CoV-2 negative in CSF, high IL-6 in CSF. IgG immunoreactivity of mouse brain tissue from serum and CSF (unknown antigen) | mild | Plasma exchange + IVMP | On day 26 and after two courses of plasmapheresis, the patient was extubated and was autonomically stable. Steroids discontinued because of insomnia and elevated mood |
| Guilmot A et al. | 80, n/a | Anti-Caspr2 encephalitis | Definite anti-Caspr2 encephalitis | isolated neurological syndrome | 3-week history of asthenia and weight loss, followed by several episodes of paroxysmal dysarthria, one tonic–clonic seizure followed by persistent neuropsychiatric symptoms (visual hallucinations, short-term memory disturbance and anxiety). No respiratory symptoms occurred | normal | Pleocytosis (9 WBC), OCB. SARS-CoV-2 negative in CSF. Antibodies anti-Caspr2-in CSF and serum | asymptomatic | IVMP, plasma exchange | n/a (initiated at the time of writing) |
| Efe IE et al. | 35, F | Encephalitis | Definite left limbic encephalitis | +14 | headache, nausea, dizziness, and drug-refractory seizures. Left anterior temporal lobectomy was performed. Postoperatively, she tested positive for COVID-19 in rt-PCR and antibody tests. Mild flulike complaints 2 weeks before onset of neurologic symptoms. On postoperative day 5, the diagnosis of encephalitis was confirmed on histopathologic examination | hyperintense signal in the left temporal lobe in T2 and T2 fluid-attenuated inversion recovery (FLAIR) imaging. Spectroscopy showed marked elevation of the choline peak along with a decrease of the N-acetylaspartate peak, suggestive of high-grade glioma | n/a | mild | n/a | n/a |
| Sarigecili et al. | 7,M | Anti-NMDAR encephalitis | Definite anti-NMDA receptor encephalitis | isolated neurological syndrome | Ataxia, somnolence and seizures, elevated CRP and lymphopenia (0.7). EEGs were encephalopathic with widespread delta waves. Day 8 of admission, choreiform movements in the hands and feet, tongue protrusion, bruxism, lip smacking, agitation, catatonia, echolalia, and tachycardia. | normal | SARS-CoV-2 negative in CSF, antibodies anti-NMDAR in CSF | asymptomatic | Plasma exchange, IVIg, IVMP | Gradual improvement within 2 weeks after steroids. Discharged mild ataxic at day 31 |
| McHattie et al. | 53, F | Anti-NMDAR encephalitis | Definite anti-NMDA receptor encephalitis | -14 | 2-week history of confusion, fever, and myalgia. Elevated CRP and lymphopenia (0.8). Naso-pharyngeal swab for SARS-CoV-2 RNA was negative at admission. Severe echolalia, palilalia, and high-pitched voice, echopraxia and behavioral disinhibition. EEG slow activity. Despite treatment with steroids, she deteriorated and developed bilateral COVID-19 pneumonia (positive for SARS-COV-2 day 14), needing mechanical ventilation. No evidence of tumor. Focal seizures and prominent dysautonomia in ICU. | hyperintensity in left amygdala and anterior putamen | Pleocytosis, SARS-CoV-2 negative in CSF, antibodies anti-NMDAR in CSF | critical | IVIg (tocilizumab for COVID19) | Great improvement at 1 month, persistence of left-side weakness. Amygdalohippocampal atropohy in follow-up MRI |
| Sánchez-Morales et al | 14, M | Anti-NMDAR encephalitis | Definite anti-NMDA receptor encephalitis | isolated neurological syndrome |  | normal | SARS-CoV-2 PCR positive in CSF (neg IgG), antibodies anti-NMDAR in CSF | asymptomatic | n/a | mRS 0 |
| Allahyari et al. | 18, F | Encephalitis | Definite anti-NMDA receptor encephalitis | simultaneous | Admitted with with generalized tonic–clonic seizures. 3-week history of mood change as depression and anhedonia accompanied by lack of concentration, low-grade fever, dry coughs, and shortness of breath. Lymphopenia. Low level of consciousness, tranfered to ICU (no need of mechanical ventilation) | brain edema in CT, MRI normal | Traumatic LP with pleocytosis, SARS-CoV-2 PCR weakly positive in CSF, CSF anti-NMDA receptor antibodies | n/a (mild-severe) | IVIg (25 g/day), IVMP (2.5 g/5 days), oral steroids taper | After 2 months of hospitalization, discharged with full recovery. |

**Table S3.** PNS immune-mediated disorders (aggregated information) of series with at least 5 patients.

| **Reference** | **N** | **Paper_inc_criteria** | **Clinical presentation** | **CSF** | **SARS-CoV-2** | **COVID severity** | **Neurophysiology** | **Treatment** | **Outcome** |
| --- | --- | --- | --- | --- | --- | --- | --- | --- | --- |
| Toscano et al | 5 | Observational case series of patients presenting with Guillain-Barre Syndrome and postive SARS-CoV-2 test | 5/5 patients presented with flaccid lower limb weakness, with 3/5 progressing to tetraparesis. All were areflexic. 3/5 patients had facial weakness and one patient had bulbar involvement. All patients had antecdent infective symptoms 5-10 days prior to onser of neurological symptoms. Antiganglioside antibodies negative in 4/4 tested. | 3/5 patients had albumincytological dissociation. WCC <5 in all five patients. SARS-CoV-2 CSF PCR negative in 5/5. | 4/5 patients had postive SARS-CoV-2 nasopharyngeal swab. The other patient had anegative swab but postive SARS-CoV-2 IgG serology at onset. | 100% of patients had mild symptomatic illness | AIDP = 2  AMAN = 1  AMSAN = 2 | 5/5 patients received IVIG. Two patients had a second IVIG cycle and another patient additionally received PLEX | 2/5 required invasive ventilation for neuromuscular respiratory failure, one patient required non-invasive ventilation.  At 4 weeks after treatment, 4 patients remained tetraplegic (2 mild improvement), 1 able to walk independently. No formal outcome scale used to measure outcome |
| Manganotti et al. | 5 | Observational case series of patients presenting with Guillain-Barre Syndrome and postive SARS-CoV-2 test over a two-month period | 4/5 presented with flaccid tetraparesis, another patient had isolated lower limb weakness, 4/5 were globally areflexic. One patient presented with bilateral opthalmoplegia, ataxia, right sided facial hypoesthesia and weakness. 2/5 had unilateral facial weakness. All patients had antecedent infective symptoms between 14 and 33 days of onset of neurology. Antiganglioside negative in 4/4 patients tested. | 3/4 had albuminocytological dissociation. WCC <5 in 4/4. SARS-CoV-2 CSF PCR negative in 4/4 | 5/5 patients had postive SARS-CoV-2 nasopharyngeal swab | 3/5 patients had cricital illness requiring mechanical ventilation. The other two patients had severe illness, requiring oxygen therapy | AIDP = 3 AMSAN = 2 | 4/5 patients received one cycle of IVIG, the other patient received 60mg methylprednisolone course over 5 days | 3/5 required mechanical ventilation due to respiratory illness rather than neuromuscular.  4/4 patients who received IVIG demonstrated improvement in weakness. No improvement was seen in the patient receiving methylprednisolone.  No formal outcome scale used to measure outcome |
| Fragiel et al. | 11 | Restrospective case-control of patients presenting through the emergency department and diagnosed with Guillain-Barre Syndrome. 61 Spanish emergency departments partook in study. Period March 1st-April 30th 2020. | Classic sensorimotor 7/11 (63.6%), pure motor 2/11 (18.2%), paraparetic 1 (9.1%), Bilateral facial palsy with parasthesias 1 (9.1%) Median of 10 days between respiratory symptoms and onset of neurological symptoms. Antigangliosides not tested | Protein >0.4g/dL 4/11 (36.4%) WCC <10 in 6/11 (54.5%)  One patient had positive SARS-CoV-2 on CSF PCR testing | 4/11 (81.8%) had postive SARS-CoV-2 PCR on nasopharyngeal swab  5/11 had postive IgG/IgM SARS CoV-2 serology  2/11 patients did not have confirmed infection on either serology or PCR | 2/11 had critical illness and required mechanical ventilation   Severity information not available for remaining patients | Information not provided | 11/11 received IVIG | 2/11 required ventilation for neuromuscular respiratory failure.  No other outcome information provided |
| Filosto et al. | 30 | Retrospective observational study involving 12 hospitals in Italy. Inclusion criteria were patients >18 with a diagnosis of Guillain-Barre syndrome fullfilling the Brighton Criteria, presenting between March and April 2020. COVID-19 postive patients and COVID-19 negative patients were then compared for any similar or differing characteristics. | Tetraparesis 83.3%, predominant paraparesis 3.3%, predominant upper limb paresis 10%, any limb paresis 3.3%. Hyporeflexia/areflexia 96.6%  Occulomotor nerve palsy, 10%, facial nerve palsy 46.7%, bulbar nerve palsy 23.3%  Onset between COVID-19 and neurological symptoms mean 24.2 days (SD +/- 11.6)  Antigangliosides not tested | Albuminocytological dissociation in 33.3%  Normal CSF 66.7% | 25/30 (83.4%) had postive SARS-CoV-2 PCR on nasopharyngeal swab. The remaining five patients had postive IgG/IgM SARS CoV-2 serology | Not enough information provided to determine | AIDP = 23 (76.6%) AMAN = 2 (6.7%) Abnormal, unclassifiable electrophysiology = 5 (16.7%) | 25/30 had IVIG 2/30 had PLEX 3/30 had no treatment | 23/30 (85.2%) of patients responded to treatment  Mixed outcome measures used. Variable time points (assessed on discharged)  5/29 (17.2%) patients required invasive ventilation and 20/29 (68.9%) required non-invasive ventilation. However it is unclear if this was due to severe respiratory illness or due to neuromuscular weakness |
| Garnero et al. | 6 | Retrospective observational study across six hospitals in Liguria region of Italy. Patients included in fulfilled Brighton Diagnostic Criteria for Guillain-Barre Syndrome and presented between 15th February and 3rd May 2020. | Information on clinical presentation only available for two patients. One patient presented with bilateral ptosis, dysphagia, bilateral X and XII CN palsy, bilateral masseter weakness, global hyporeflexia, no limb weakness. The other patient had acute four limb weakness with distal predominance and asymmetry to right leg. Dysautonomia with paralytic ileus and liable BP  Antecedent illness between 0 and 20 days of onset of neurological symptoms  Antiganglioside antibodies negative in 6/6 | Albuminocytolgical dissociation present in 2/4 patients.  SARS-CoV-2 CSF PCR negative in 4/4 | 6/6 patients had postive SARS-CoV-2 PCR on nasopharyngeal swab | Two patients had critical illness requiring mechanical ventilation.  Information not available on COVID severity for 4/6 patients | AIDP = 1 AMSAN = 2 MFS = 1 Electrophysiology not available = 2 | All treated with IVIG | Improvement in MRC sum score and GBS disability score seen in 4/6 patients (average 26 day follow up). The other two patients showed deterioration in both scores |
